# Precipitating Factors for Acute Heart Failure Hospitalization and Long-Term Survival

**DOI:** 10.1097/MD.0000000000002330

**Published:** 2015-12-31

**Authors:** Anat Berkovitch, Elad Maor, Avi Sabbag, Fernando Chernomordik, Avishay Elis, Yaron Arbel, Ilan Goldenberg, Ehud Grossman, Robert Klempfner

**Affiliations:** From the Leviev Heart Center (AB, EM, AS, FC, IG, RK); Department of Internal Medicine D (AB, EG); Pinchas Borenstein Talpiot Medical Leadership Program (EM); all at the Chaim Sheba Medical Center; Sackler School of Medicine, Tel-Aviv University (AE, IG, EG, RK); Department of Internal Medicine C, Beilinson Hospital, Rabin Medical Center (AE); and Cardiology Department, the Tel Aviv Sourasky Medical Center (YA), all are affiliated to Sackler School of Medicine, Tel-Aviv University.

## Abstract

Heart failure (HF) patients have frequent exacerbations leading to high consumption of medical services and recurrent hospitalizations.

Different precipitating factors have various effects on long-term survival.

We investigated 2212 patients hospitalized with a diagnosis of either acute HF or acute exacerbation of chronic HF. Patients were divided into 2 primary precipitant groups: ischemic (N = 979 [46%]) and nonischemic (N = 1233 [54%]). The primary endpoint was all-cause mortality.

Multivariate analysis demonstrated that the presence of a nonischemic precipitant was associated with a favorable in-hospital outcome (OR 0.64; CI 0.43–0.94), but with a significant increase in the risk of 10-year mortality (HR 1.12; CI 1.01–1.21). Consistently, the cumulative probability of 10-year mortality was significantly higher among patients with a nonischemic versus ischemic precipitant (83% vs 90%, respectively; Log-rank *P* value <0.001). Subgroup analysis showed that among the nonischemic precipitant, the presence of renal dysfunction and infection were both associated with poor short-term outcomes (OR 1.56, [*P* < 0.001] and OR 1.35 [*P* < 0.001], respectively), as well as long-term (HR 1.59 [*P* < 0.001] and HR 1.24 [*P* < 0.001], respectively).

Identification of precipitating factors for acute HF hospitalization has important short- and long-term implications that can be used for improved risk stratification and management.

## INTRODUCTION

Heart failure (HF) is an increasingly prevalent disease in developed countries.^1^ Population-based studies have estimated that ∼1 to 2% of the adults in developed countries have HF, with the prevalence rising to >10% among persons 70 years of age or older.^2^ This population is expected to increase ^3^ as the survival rates of both ischemic heart disease (IHD) and HF are improving.

During the past decade, there has been a stepwise improvement in terms of understanding the pathophysiology and treatment of chronic HF patients. Despite better medical care, HF patients are frequently re-hospitalized and consume medical services.^4^ In fact, HF is considered to be the number 1 reason for readmission in both medical and surgical groups.^5^ Therefore, it is important to identify factors associated with long-term morbidity and mortality among patients hospitalized for acute HF. Precipitating factors leading to heart failure decompensation can be identified in many instances when a careful history is obtained,^6–8^ and may be associated with subsequent outcomes. Precipitants represent important targets for preventive interventions that are highly cost effective. However, limited information exists regarding the long-term implications of common precipitating factors and whether different precipitants exert a differential long-term effect on outcomes. It is possible that correctly identifying those factors can assist both short- and long-term evaluation and management practices and potentially improve outcomes. Accordingly, the present study was carried out in a large cohort of patients enrolled in the Heart Failure Survey in Israel (HFSIS) and was designed to assess: (1) the frequency of specific precipitants for acute HF admission in a real-world setting; and (2) the implications of commonly identified precipitants on short- and long-term survival in this population.

## METHODS

### Patient and Data Collection

The design and methods of the HFSIS have been described previously.^9,10^ Briefly, the survey, conducted in March 2003 to April 2003, included 4102 patients diagnosed with either acute de novo, acute exacerbation, or chronic HF, who were admitted to 93 of the 98 internal medicine departments and 24 of the 25 cardiology departments in all 25 public hospitals in Israel. At admission, all patients underwent chest radiography and blood tests in addition to the physical examination. The criteria used for diagnosing HF were symptoms of HF (at rest or during exercise) and objective evidence of cardiac dysfunction at rest. Acute HF was defined as a rapid onset or change in the signs or symptoms of HF, resulting in the need for urgent therapy. Acute HF may be secondary to either acute de-novo HF or acute exacerbation of chronic HF. Of the total 4102 patients, 1890 were hospitalized with a diagnosis of chronic HF without an acute exacerbation and were therefore excluded from the analysis. Thus, the final study population comprised 2212 patients who were hospitalized with a diagnosis of either acute de novo or acute exacerbation of chronic HF. In-hospital complications and mortality data were collected by study personnel using prespecified forms. One-year as well as 10 years mortality data were ascertained through the Israeli National Population Registry. The protocol was approved by the respective Ethics Committee at each of the participating hospitals.

### Collection of Data Regarding Precipitating Factors

Precipitating factors were prospectively collected using a predefined form. More than 1 precipitating factor was allowed and precipitants selection was based on the clinical judgment of the treating physician following emergency room or department investigations. Available options describing the main precipitants were: arrhythmia (according to documented electrocardiogram), myocardial ischemia (defined as any acute coronary event), valvular pathology (according to echocardiography or clinical findings and history), thyroid dysfunction, excessive intake of fluid, salt or alcohol excess, medication non-adherence, medication related adverse events, anemia, pulmonary emboli, renal dysfunction (acute or exacerbation of chronic kidney dysfunction), infection, physical or mental stress (according to the patient's declaration), documented cerebrovascular event (CVA) or transient ischemic attack (TIA), and diabetes mellitus (DM) related hypoglycemia (defined as glucose level <70 mg/dL with appropriate symptomatology) or hyperglycemia (defined as glucose level >350 mg/dL) event.

### Definitions and Outcome Measures

The presence of DM was defined by one of the following criteria: a history of DM obtained from medical records, admission blood glucose ≥200 mg/dL, or the use of antidiabetic agents (on admission or discharge). NYHA functional class was determined according to functional status and symptoms before index hospitalization. Renal function was categorized using MDRD formula for the estimated glomerular filtration rate (eGFR) as follows: good renal function ≥60 mL/min/1.73 m^2^, and impaired renal function eGFR < 60 mL/min/1.73 m^2^. Anemia was defined as hemoglobin <11 mg/dL, and clinically significant hyponatremia as plasma sodium <130 meq/dL, on the admission laboratory tests.

For the present study, participants were categorized into 2 main groups according to the specific cause for the exacerbation: ischemic and nonischemic (including all other precipitating factors listed above). If >1 precipitant was chosen at admission, and one of the precipitants was “myocardial ischemic,” the patient was included in the ischemic group. In subjects with suspected ischemia precipitant biomarkers were obtained. In a secondary analysis we further categorized nonischemic precipitants into the following groups: infection, renal dysfunction, noncompliance with medication, and other precipitating factors.

The short- and long-term primary outcome measures of the present study included all-cause mortality during the index hospitalization and at 10-years of follow-up, respectively. Every effort was made to ensure accurate and reliable profiling data, which included standardizing HF and data validation definitions.

## Statistical Analysis

Baseline characteristics of the patients were compared by grouping them into the 2 main precipitant categories. Comparison of categorical variables was performed with chi-square analysis and comparison of continuous variables was performed with the Student's *t* test for variables with normal distribution and by Kruskal–Wallis for those that violated the normality assumption.

Cumulative probability of 10-year mortality curves according to 2 main precipitating factor categories was constructed according to the Kaplan–Meier method and compared using the log-rank test. An additional Kaplan–Meier estimate was constructed for the comparison among the specific nonischemic precipitants.

Logistic regression analysis, comparing the 2 main groups (ischemic and nonischemic), was performed in order to identify independent predictors for in-hospital mortality. The multivariate model included the additional prespecified covariates: age, gender, smoking status, past MI, LVEF < 50%, NYHA III-IV, hypertension, dyslipidemia, DM, and renal dysfunction. A secondary analysis was performed in a similar manner, substituting the primary nonischemic precipitant group with the 4 major nonischemic subcategories (infection, renal dysfunction, noncompliance with prescribed medication, other) each compared with the ischemic precipitant as the reference group. Similarly, multivariate Cox proportional-hazards regression analysis was carried out to assess factors independently associated with 10-year mortality, including the same prespecified covariates. Proportionality of hazard in the described regression models was verified according to the log minus log method (LML).

All *P* values were 2 sided, and a *P* value ≤0.05 was considered significant. The statistical software used was SPSS version 20 (IBM Inc).

## RESULTS

### Study Population Characteristics

The present study population was comprised of 2212 patients hospitalized with acute HF with a mean age of 75 ± 10 years, of whom 1214 (55%) were men. Precipitating factors were grouped into the 2 main prespecified categories, ischemic and nonischemic precipitants. An acute ischemic precipitant was identified in 979 (46%) individuals. The majority of patients with an ischemic precipitant (n = 979) had a non-ST elevation AMI (n = 139; 14%) or angina pectoris (n = 694; 71% of which only 249 had unstable angina pectoris). Only 146 (15%) had an STEMI thus primary reperfusion was infrequent (primary PCI n = 104, thrombolysis n = 36). The rate of coronary revascularization in ACS patients presenting without ST-elevation was 78%. In subjects with suspected ischemic precipitant, troponin was obtained in 318 (14%) patient and CPK in 1314 (59%), whereas 70 subjects had no available biomarker result. The major nonischemic precipitants were infection (21%), noncompliance (17%), renal dysfunction (13%), and other miscellaneous factors (49%). The baseline clinical characteristics of study patients categorized by the main precipitating factor categories are presented in Table 1. Patients with an ischemic factor as a precipitant for the acute HF hospitalization were younger compared to the nonischemic group and more likely to be men. In addition, patients in the ischemic group were more likely to have dyslipidemia (44%), DM (47%), left ventricle dysfunction (64%), and to have had previous myocardial infarction ([MI] 48%), but were less likely to have NYHA (New York Heart Association) functional class III or IV (39%), anemia (26%), and their BMI was lower compared to the nonischemic group (27 kg/m^2^ vs 28 kg/m^2^) (Table 1). Other baseline characteristics during the index hospitalization, including serum sodium, renal function, and history of CABG (coronary artery bypass graft) did not differ significantly between the 2 main precipitant groups.

**TABLE 1:**
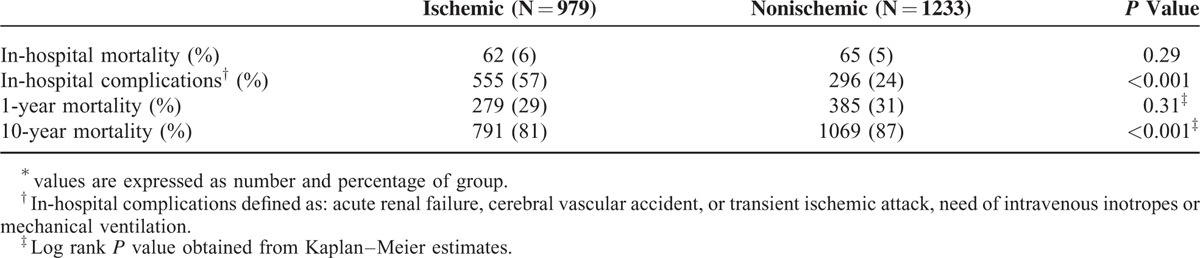
Baseline Characteristics of Study Population by Precipitating Factors Groups

### Survival, Crude Mortality, and Complication Rates

Median survival for the entire study population was 32.6 months (±1.3). Patients with ischemic precipitant had higher median survival compared with the nonischemic group (38.4 vs 29.6, respectively). In the nonischemic group, patients in the other precipitants group had the highest median survival (35.3), followed by noncompliance (30.9), renal dysfunction (22.1), and infection (21.6).

Data regarding in hospital complications, short- and long-term mortality rates are presented in Table 2. Patients with an ischemic precipitant for the index HF hospitalization were shown to experience a significantly higher rate of in-hospital complications compared with those who had a nonischemic precipitant (57% vs 24%; *P* < 0.001), whereas crude in-hospital and 1-year mortality rates were similar between the 2 main precipitant groups. In contrast, at 10-years of follow-up crude mortality rates were higher among patients who had a nonischemic precipitant for the index hospitalization as compared with those with an ischemic precipitant (87% and 81%, respectively).

**TABLE 2:**
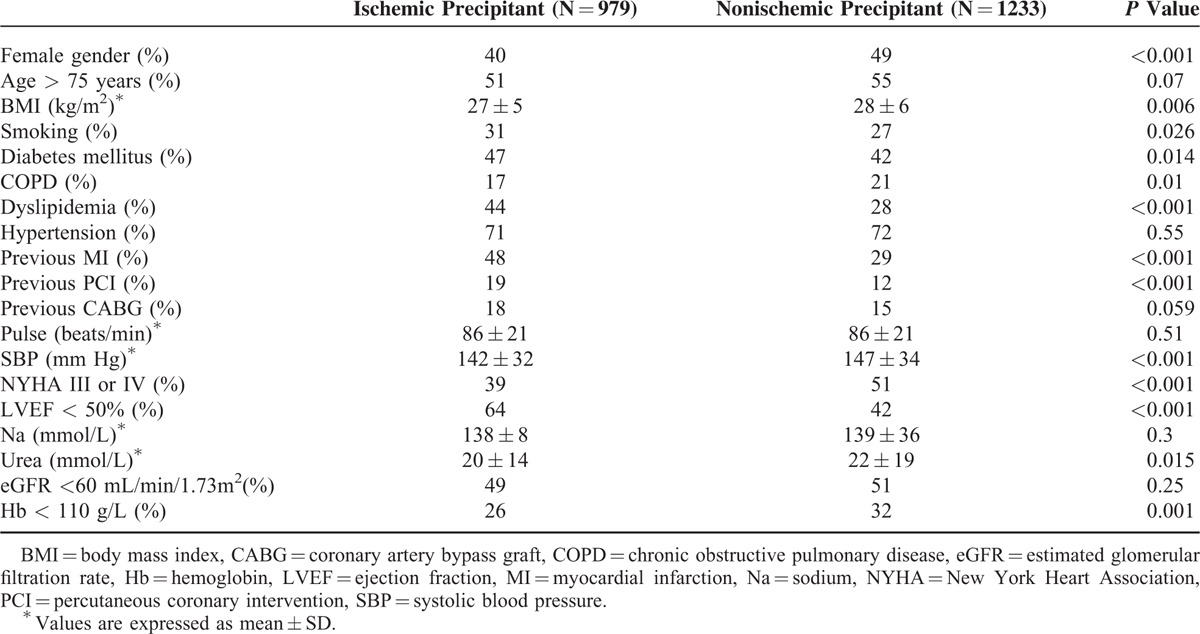
Crude Event Rates for the Prespecified Outcomes^∗^

**FIGURE 1:**
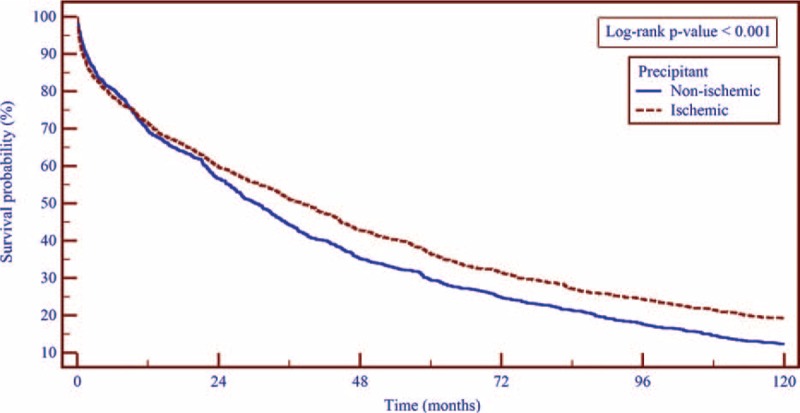
Kaplan–Meier curves showing the survival cumulative probability according to the ischemic and nonischemic principle precipitants grouping. The analysis illustrates the cumulative survival probability according to the cause of HF exacerbation. Log-rank *P* value <0.001.

Consistent with these findings, Kaplan–Meier survival analysis showed that during 10-year of follow-up, mortality rates were lower among patients who were hospitalized with an ischemic precipitant compared with those who were hospitalized with a nonischemic precipitant (83% vs 90%; log-rank *P* value < 0.001; Figure 1), with evidence of separation in event rates between the 2 groups after ∼10 months following the index hospitalization.

### Multivariate Analysis: Independent Predictors for In-Hospital and 10-Year Mortality

Factors independently associated with increased risk for in-hospital and 10-year mortality among study patients are shown in Table 3. After multivariate adjustment, the presence of a nonischemic precipitant for the index hospitalization was shown to be independently associated with reduced risk of in-hospital mortality (OR 0.64, CI 0.43–0.94, *P* = 0.025). In-contrast, multivariate modeling for 10-year mortality showed a risk-reversal whereas the presence of a nonischemic precipitant was shown to be independently associated with a significant 12% increased (HR 1.12, CI 1.01–1.23, *P* value = 0.03) risk of 10 years all-cause mortality compared to the ischemic group. Additional factors shown to be independently associated with increased risk for both in-hospital and 10-year mortality included age, NYHA III-IV, and renal dysfunction (Table 3). Similar results were obtained when mortality risk was calculated for 4 years.

**TABLE 3:**
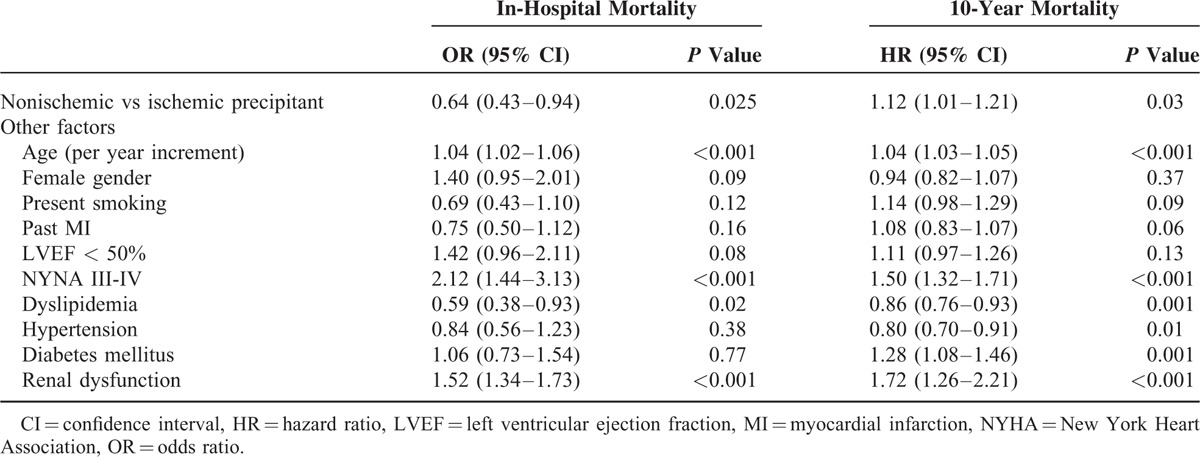
Multivariate Analysis: Risk Factors for In-hospital and 10-Year Mortality

An additional analysis comparing acute on chronic versus acute de novo heart failure was performed. Long-term mortality risk was reduced when new onset heart failure was compared to exacerbation of chronic heart failure (HR 0.77; CI 0.69–0.86) following adjustment for age, gender, NYHA class, eGFR, sodium, BUN, and LVEF. Consistent results were obtained when the HF onset type was added to our multivariate model.

### Subgroups Analysis: Association Between Specific Nonischemic Precipitants and Outcomes

**FIGURE 2:**
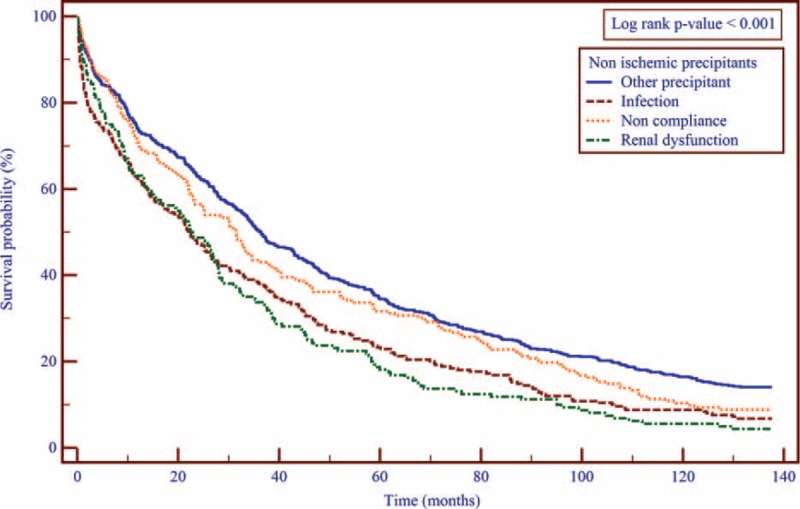
Kaplan–Meier curves showing the 10-year survival cumulative probability according to the major nonischemic subtypes. Log-rank *P* value <0.001.

Kaplan–Meier survival analysis (Figure 2) showed that of the nonischemic precipitants, patients with renal dysfunction had the highest mortality rates at 10 years (96% mortality rates), followed by infection (93% mortality rates), and noncompliance (91% mortality rates). Notably, 10-year mortality among patients with an ischemic precipitant was lower compared with each of the individual nonischemic precipitants that were assessed.

Consistent with these findings, multivariate analysis showed that renal dysfunction and infection, as precipitants, was independently associated with increased risk for both short- and long-term mortality (Table 4). Compared with patients who had an ischemic precipitant for the index hospitalization those who had renal dysfunction experienced a significant 56% (*P* < 0.001) increased risk for in-hospital mortality and a 59% (*P* < 0.001) increased risk for 10-year mortality. Similarly, compared with an ischemic precipitant, infection was associated with a significant 35% (*P* < 0.001) increased risk for in-hospital mortality and a 24% (*P* < 0.001) significant increased risk for 10-year mortality. Notably, noncompliance with medication as a precipitant was associated with reduced in-hospital mortality compared to an ischemic precipitant, yet significantly increase the 10-year mortality risk compared to ischemic precipitant (OR 0.22 CI 0.08–0.62 and HR 1.32 CI 1.14–1.53, respectively). Similar results were obtained when mortality risk was calculated for 4 years.

**TABLE 4:**
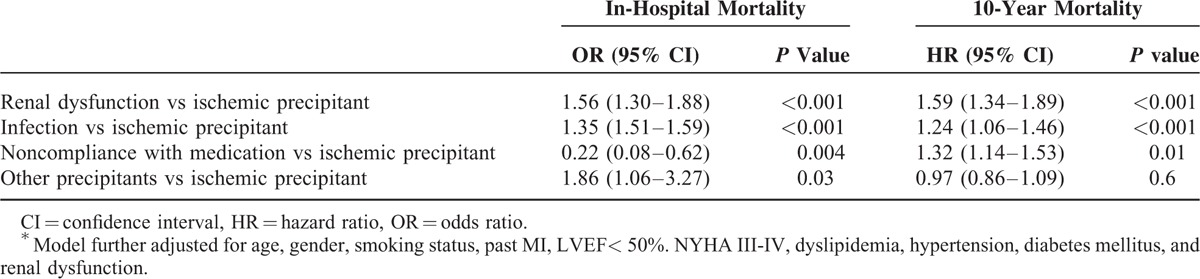
Subgroups Analysis: Association Between Individual Nonischemic Precipitants and Mortality^∗^

Finally, we recalculated the regression models using covariates from the manuscript by Cleland et al^11^ and obtained similar results. Nonischemic participants had a significant 11% higher 10-years mortality risk compared with the ischemic group. In the subgroup analysis, patients with low-compliance and infection had higher 10-years mortality risk (23% and 27%, respectively). However, renal dysfunction and other participants lost statistical significance.

## DISCUSSION

The present study provides several important implications regarding risk assessment among patients hospitalized with acute HF. We have shown that: (1) nearly half of the patients who are hospitalized with acute HF in a real-world setting have an ischemic precipitant for the event; (2) the identification of specific precipitants has important short- and long-term implications in this population, wherein acute ischemic as a precipitant factor is independently associated with increased in-hospital mortality, but with improved long-term survival; and (3) the main factors affecting long-term survival following hospitalization with acute HF are nonischemic precipitants, including renal dysfunction, infection, and noncompliance with prescribed medication alongside well-established predictors such as DM, NYHA functional class, smoking status, and age.

### Influence of Different Precipitating Factors

Our findings stand in contrast to those of Chun et al,^12^ whose study followed HF patients for 10 years after index hospitalization and concluded that patients admitted for ischemic reasons have a higher mortality rate than other HF patients. One explanation of this discrepancy might be the provision of better primary care to such patients. In addition, some of these patients continue to have closely regulated medical care and engage in heart rehabilitation, which was found to improve clinical outcomes in previous papers.^13,14^ Diaz et al^7^ investigated 102 cases of HF and found noncompliance with medications and diet to be the most frequent reason for exacerbation and hospitalization of HF patients, followed by ischemic reasons. However they did not address long-term survival and had a small sample size. In the paper of Gheorghiade et al,^15^ a thorough literature review was conducted regarding hospitalization for heart failure. Nevertheless, little attention was given to precipitating factors and no long-term mortality rates were mentioned. Finally, Opasich et al^16^ identified precipitating factors for HF destabilization and found poor compliance and infection to by most frequent, followed by MI. These differences are probably due to the fact that Opasich addressed all HF destabilization, whereas we examined only those that were hospitalized. Renal function deterioration was found to be a significant precipitating factor in all follow-up intervals. This supports previous reports that have found worsening renal function to be associated with longer lengths of stay, higher in hospital costs, increased in-hospital mortality, and greater likelihood of readmission.^17^

Infection, as a cause of exacerbation, was found to be both short-term and a long-term poor prognostic factor. This finding strengthen the paper published by Alon et al,^18^ who found that HF patients hospitalized for infectious reasons had higher mortality rates at 30 days and 1 year. They concluded that infection is both a precipitator for decompensation of HF as well as a direct contributor for mortality. The reason for a higher in hospital mortality is clear, but the reason that an isolated event effects long-term survival is still unclear. Previous studies have shown that patients hospitalized with pneumonia have increased long-term mortality rates.^19^ In addition, a recent paper ^20^ have found pneumonia hospitalization to be an independent risk factor for long-term cardiovascular events, and a tribute this increased risk to pro-inflammatory changes. We hypothesize that these patients have a weakened immune system, greater comorbidity burden, and may also suffer from suboptimal medical care, compared to patients having ischemic event. Our ability to address, treat and care for ischemic patients is far better than our treatment solutions regarding renal dysfunction or a weak immune system.

Renal dysfunction is a well-known independent prognostic factor for poor long-term outcomes in patients with HF exacerbations.^21^ The cause and mechanisms for the worsening renal function among HF patients are probably multifactorial, including impaired cardiac output and under perfusion of renal arteries, venous congestion, and elevated intra-abdominal pressure.

Compliance with medications as well as compliance with nonpharmacologic recommendations is an issue investigated previously, as a preventable causative factor for HF exacerbation.^22–24^ In this current study, noncompliance with medications had a better short-term outcome but was found to have a negative long-term prognostic factor. We can assume that patients admitted for HF exacerbation as a consequence of noncompliance have a somewhat better short-term outcome because it is an easy precipitant to address, as patients hospitalized are subject to regular eating and medication regimens which are supervised by the medical staff. Hospitalization neutralizes factors that may contribute to noncompliance such as financial issues that affect the availability of food and medicine and forgetfulness. Therefore it is not surprising that many of these same patients return to their “bad habits” ^25^ and eventually have worse long-term outcome.

According to our analysis there are a number of precipitating factors that may differently influence the short- and long-term survival of patients having HF exacerbation. These factors, some of which are subject to change, should be considered and addressed by the physician in every visit following discharge. We encourage physicians to have more close follow-up in these cases and recognize the early signs of deterioration. We also found that the impact of each precipitating factor may change over time.

## CONCLUSIONS

We have shown that ischemia, as a precipitating factor for HF exacerbation, is associated with somewhat better long-term survival compared to nonischemic factors such as infection, renal function, and noncompliance with medications. These findings suggest that every attempt should be made to identify and address these factors in order to improve risk stratification and management strategies in this high-risk population.

## LIMITATIONS

The main strengths of this study were the large sample size from a national survey and the substantial variety among patients that allows physicians to apply these results to a large population. In addition, the patients in this study represent the population of patients that doctors encounter every day. However, due to the fact that patients often present with >1 precipitant factor this so-called limitation is also one of the study's advantages, as each precipitant's presence was accounted for by the presence of any others, thus representing the independent influence of outcomes. In addition, we do not have data regarding the postdischarge management or the actual cause of death.
